# Screening for hepatitis D and PEG-Interferon over Tenofovir enhance general hepatitis control efforts in Brazil

**DOI:** 10.1371/journal.pone.0203831

**Published:** 2018-09-07

**Authors:** Ashish Goyal, Ethan Obie Romero-Severson

**Affiliations:** Theoretical Biology and Biophysics, Los Alamos National Laboratory, Los Alamos, New Mexico, United States of America; Centre de Recherche en Cancerologie de Lyon, FRANCE

## Abstract

**Background:**

Hepatitis D virus (HDV), which requires the presence of hepatitis B virus (HBV), is a deadly yet neglected disease that rapidly leads to liver cancer and disease-induced mortality. This co-dependence creates complex transmission dynamics that make it difficult to predict the efficacy of interventions aimed at HBV and/or HDV control in endemic regions, such as certain municipalities of Brazil, where up to 65% of HBV-infected persons are co-infected.

**Methodology:**

We created a mathematical model that captures the joint transmission dynamics of HBV and HDV, incorporating mother-to-child, sexual and household transmission. With an aim to minimize the number of total infections and disease-induced mortality in 2027, we then determined optimal strategies for Brazil and its sub-regions under a constrained budget, which was dynamically allocated among HBV and HDV screening, HBV and HDV treatment, HBV newborn and adult vaccination, and awareness programs. Three treatment options were considered, namely: Tenofovir, PEGylated-Interferon, and nucleic acid polymers (NAP).

**Results:**

The additional cost of HDV screening and the use of a more expensive PEGylated-Interferon are offset by not wasting resources on treating co-infected persons with Tenofovir. The introductory price of NAP treatment must be less than $16,000 per course to become competitive with Tenofovir and PEGylated-Interferon in Brazil.

**Conclusion:**

Additional screening for HDV is beneficial, even in a low HBV and HDV endemic regions of Brazil. We recommend PEGylated-Interferon, wherever possible, for both HBV and HDV. If PEGylated-Interferon is not available in abundance, PEGylated-Interferon for co-infections and 4-year Tenofovir treatment for mono-infections is recommended.

## Introduction

Together, hepatitis B virus (HBV) and hepatitis D virus (HDV) are a major global health burden with approximately 240 million infections worldwide [1]. Although HBV itself is a major chronic illness that leads to liver cirrhosis, hepatocellular carcinoma, and death, co-infection with HDV increases disease progression rates by 5–10 fold. Therefore, the presence of HDV significantly amplifies the morbidity and mortality of existing HBV epidemics and negatively affects the performance of interventions aimed towards HBV eradication [2–4].

The majority of chronic HBV carries reside in developing or under-developed regions such as Asia Pacific and sub-Saharan Africa, where chronic HBV infection is highly endemic (>8% prevalence). Intermediate HBV endemic regions have 2–7% chronic HBV prevalence and include regions such as North Africa and the Middle East, parts of Eastern and Southern Europe, parts of Latin America, and South Asia. More developed regions/countries such as Australia, Asia, Northern and Western Europe, Japan, North America, and some countries in South America represent low endemic regions, where chronic HBV is prevalent in less than 2% of the population [5]. Unlike HBV, there is no clear classification of HDV prevalence due to lack of our knowledge of HDV epidemiology in different regions of the world [6]. As a matter of convention, we define low, moderate, and high-endemic HDV infection as ≥8%, 2–7% and <2% HDV prevalence in HBV infected population, respectively.

In 2017 Brazil had approximately 1.5 million HBV carriers [7], of which about 8% were co-infected with HDV [8]. The distribution of HDV co-infection is spatially heterogeneous, reaching as high as 65% in some parts of Brazil such as the Amazon Basin [9–11]. Brazil constitute of regions that range from both high to low-endemic HBV and high to low-endemic HDV. Effective intervention to reduce HDV prevalence is complicated by the fact that there is no direct vaccination or effective treatment for HDV [12–14]. However, HDV can only infect person already infected with HBV, raising the possibility of indirect prevention methods by treating either HBV-only or co-infected persons [15]. Despite being deadly and widespread in some populations, HDV infections are largely neglected as a public health intervention target [14].

In 2002 [16] the Brazilian government implanted a newborn HBV vaccination program that reached over 90% by 2015 [17]. However, coverage is heterogeneous remaining on the lower side (69–87%) in the Amazon Basin [18]. Extremely high HDV prevalence is currently limited to the Amazon Basin, but it still poses a huge health risk to the rest of the country and broader region as a consequence of an increased migration to and from the Amazon Basin [19]. Over the past decade, the Brazilian government has also incorporated free HBV testing in HIV centers and universal access to hepatitis treatment in its public health agenda [20]. However given limited public health budgets, these additional measures are unlikely to have a large effect on HBV and HDV prevalence [20]. The economic burden of universal treatment for all diagnosed HBV (and HDV) infections would be substantial, especially if unaware HBV and HDV carriers continuously produce new infections. In this case, it becomes important to, (i) diagnose unaware HBV infections [21], (ii) diagnose unaware HDV infections, (iii) treat diagnosed HBV infections [21], (iv) treat diagnosed HDV infections, (v) increase newborn vaccination coverage, and (vi) spread awareness in the population to prevent the incidence of new infections. However, given limited public health resources, it is impossible to apply all these interventions at the same time. Optimal triage of limited public health resources in Brazil is essential for limiting the spread of HDV.

We implemented a mathematical model of joint HBV and HDV epidemics in Brazil accounting for age structure, mother-to-child transmission, household transmission and horizontal transmission to study the efficacy of alternative investment strategies. Mathematical modelling assists public health policy-makers in making informative decisions [22–24] and formal models are especially important in complex, multi-pathogen systems such as HBV and HDV [2–4], where intuitive reasoning may be insufficient. The model we use in this analysis is an extension of previously published work on HDV in China [25]. Unlike China, Brazil is a low HBV endemic region with sub-regions of moderate and high HBV and HDV prevalence but the control of HDV in Brazil is still more difficult due to increased burden of disease and reduced public health resources [16, 25]. We find the dynamic allocation of resources year-to-year between different controls (screening, vaccination, treatment, and awareness) under different treatment policies that reduce new cases of HBV and mortality from 2017–2027.

## Material and methods

### Model structure

**Fig 1:**
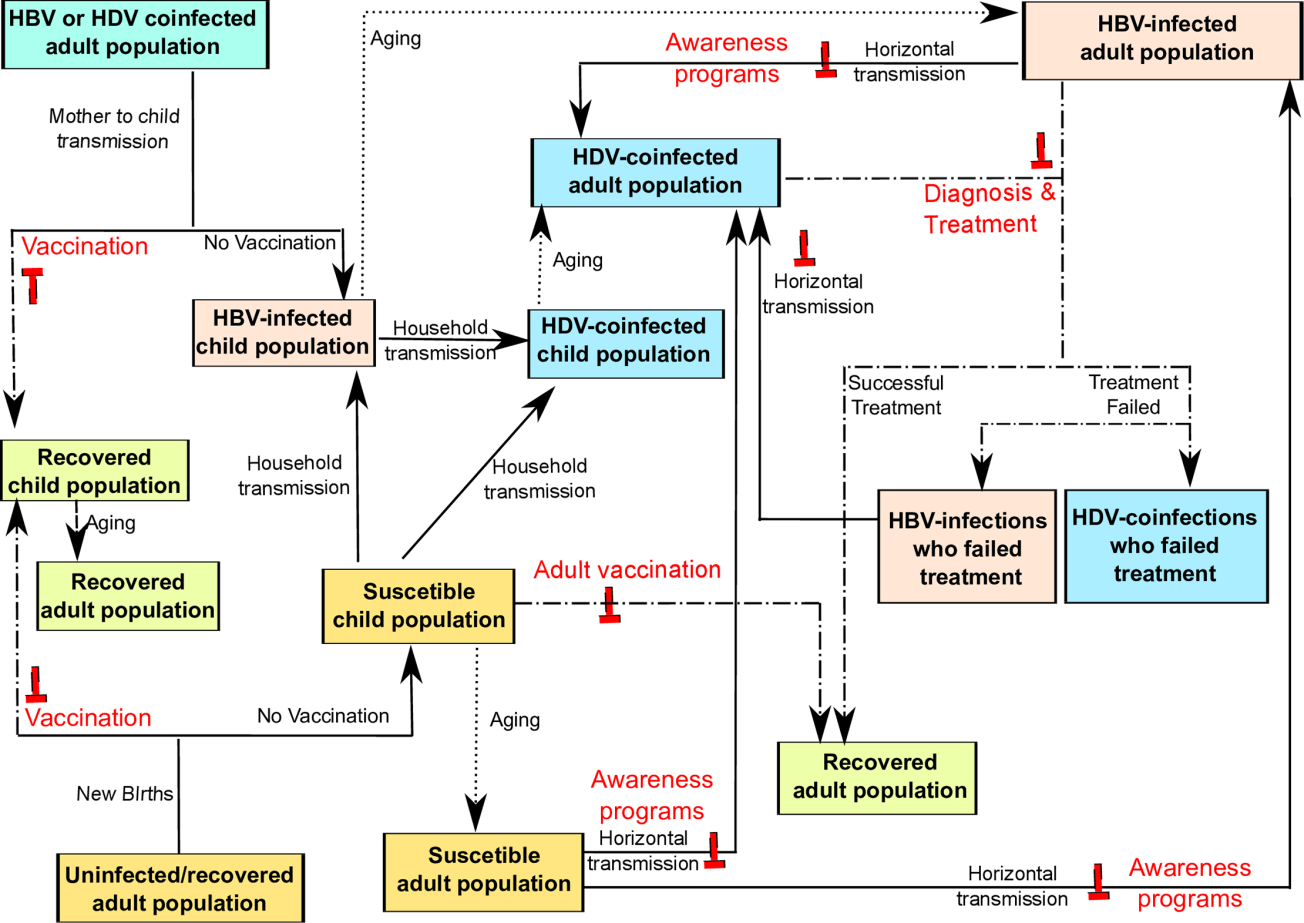
Schematic representation of HBV and HDV transmission in a population along with the interventions employed in the model to counter HBV and HDV epidemics. Green, orange, pink and blue boxes represent recovered, susceptible, HBV mono-infected, and HBV-HDV co-infected groups in the population. Text in red represent one of the five interventions applied: (i) HBV newborn vaccination (ii) HBV diagnosis and adult vaccination, (iii) antiviral treatment for HBV infected individuals, (iv) antiviral treatment for HBV and HDV infected individuals, and (v) awareness programs.

The model is represented by a system of 22 ordinary differential equations (see section A in S1 File). The population consists of two different age groups, (i) child (age≤14 years), and (ii) adult/sexually-active (age>14 years). These two age groups were categorized into four main compartments, (i) susceptible individuals; (ii) HBV mono-infected individuals, (iii) HBV-HDV co-infected individuals, and (iv) recovered individuals who are immune to future mono- and co-infections. Both mono- and co-infected women transmit HBV infection perinatally but not HDV [26]. If vaccinated at birth, newborns acquire immunity against HBV and HDV. Mono- and co-infected adults also contribute to new HBV and HDV infections in the adult population through horizontal (i.e. sexual) transmission. Furthermore, susceptible children can also acquire HBV and HDV infections through (non-sexual) interactions with infected persons (both adults and children) though household transmission [27]. The model structure is illustrated in Fig 1.

### Model calibration

We identified population parameters (Table A in S1 File) and treatment costs (Table 1) from the literature. The prevalence of HBV and HDV in both age groups (Table 2 and section D in S1 File) at the beginning of each simulation were also identified from surveys conducted in Brazil.

**Table 1:** Cost (in US dollars) and efficacy of five interventions in 2017.

Description	Value	Reference
Efficacy of newborn HBV vaccination	95%	[28–30]
Efficacy of adult HBV vaccination	95%	[31]
Awareness programs efficacy	0.5	[2]
Antiviral therapy efficacy	0.1 vs 0.1^a^ (48 week Peg-Interferon in mono-infected vs co-infected) 0.032 vs 0^a^ (1 year Tenofovir in mono-infected vs co-infected) 0.075 vs 0a (4 year Tenofovir in mono-infected vs co-infected)	[32–35]
3-dose new-born HBV vaccination cost	$3.77/person^b^	[36, 37]
The testing cost of either HBV or HDV	$3.37/person^b^	[38]
3-dose adult HBV vaccination cost	$4.08/person^b^	[36, 37]
Cost of antiviral therapy	$8172.34/person/year (48 weeks Peg-Interferon) $934/person/year (1 year Tenofovir)	[39–41]
Awareness programs cost	$0.2/person/year^b^	[2]
Life-time cost of a HBV or HDV infection	$5000^b^	

^a^ HBsAg seroconversion without relapse is being considered as an indicator of sustained virological response [33, 35, 42, 43]. The year 2, 3 and 4 efficacies of Tenofovir treatment was assumed 0.014, 0.014 and 0.014 respectively.

^b^ The medical costs in Brazil are approximately 12 times less compared to the US [36]. Therefore, wherever costs were not available for Brazil, we assumed them to be 1/12^th^ of medical costs in the US. The costs reported here are inclusive of both medical and non-medical costs as well as the follow-up costs. The treatment costs do not include cost of severe cases of HBV and HDV infection such as hepatocellular carcinoma that often requires liver biopsies and liver transplantation.

**Table 2:** Population Stats in Brazil and its sub-regions.

Region	Population in 2017^#^	(%) HBV prevalence in adults^#^	(%) HBV prevalence in children^#^	(%) HDV prevalence in HBV infected adults^#^	(%) HDV prevalence in HBV infected children^#^	Health budget in USD/ person *
Brazil	225×10^6^	0.6 [7]	1.8 [44]	8 [8]	0^a^	0.16 [16, 45, 46]
State of Acre	0.83×10^6^	3.3 [47]	1.8^b^	65 [47]	7.7^c^	0.57 [16]
Manaus	2.2×10^6^	6 [7]	1.8^b^	27 [48]	7.7^c^	0.57 [16]
Lábrea Municipality	45×10^3^	8 [49–51]	8 [49–51]	15.2 [51]	7.7[51]	0.57 [16]
Eirunepé city	35×10^3^	4.7 [48]	1.8^b^	47 [48]	7.7^c^	0.57 [16]

Initial HBV and HDV prevalence in children and adults as well as public health budget (in USD) per person in 2017 in in Brazil at the national level and its sub-regions, namely: State of Acre, Manaus, Lábrea Municipality and Eirunepé city.

^#,*^ More explanation is provided in the supplementary text (see sections D and E in S1 File).

^a^ Household transmission was neglected at the national level in Brazil but was included in the model for its sub-regions in the Amazon Basin (i.e,. State of Acre, Manaus, Lábrea Municipality and Eirunepé city).

^b^ The data was not available and therefore, we assume it to be similar as at nation-wide level.

^c^ The data was not available and therefore, we assume it to be similar in sub-regions in the Amazon Basin.

### Intervention model

Our model is inclusive of following five interventions (also illustrated in Fig 1),

- Implementation of HBV and/or HDV infection screening/diagnosis and HBV adult vaccination,
- Implementation of antiviral treatment for mono-infected individuals,
- Implementation of antiviral treatment for co-infected individuals,
- Implementation of awareness programs,
- Increase in newborn vaccination coverage from the baseline coverage of 95% and 80% coverage in Brazil and the Amazon Basin, respectively [18, 36].

The diagnosis process has two phases. In the first phase, a person is tested for HBV and, if positive for HBV, then tested for HDV. If they are positive on both tests, then they are immediately considered a candidate for antiviral treatment. However, if the person comes out as HBV positive but HDV negative, then the medical recommendation is to re-test in 6 months to determine true HBV status [2]. In the second phase, persons with a previous positive HBV test are re-tested for both infections. If the HBV test or both come out positive, then a person is immediately considered a candidate for antiviral treatment as a mono- or co-infected individual respectively. In either phase of testing, if an adult is determined to be both uninfected and unvaccinated, we assume they are given HBV adult vaccination to induce future immunity to both infections and are not tested further. HBV positivity is confirmed by the presence of either HBsAg or HBV DNA while an individual is additionally confirmed positive for HDV through HDV RNA [25]. In the model, we further assumed that 5%(1–10%) of the adult population can be diagnosed every year, based on the assumption that each of the >7000 hospital can test 1–10 person every day [52].

Diagnosed infections, as they become aware of their infection status, if not receiving treatment are assumed to transmit infections at a lower rate compared to undiagnosed infections [25]. On the other hand, diagnosed infections under treatment are assumed to be not transmitting for the treatment duration because of behavioral changes and huge suppression in HBV DNA levels in individuals resulting from treatment [15]. Furthermore, infected individuals who fail treatment are assumed to transmit infections at the same rate as diagnosed infections without treatment. The awareness programs promoting safer sex and use of condoms are assumed to induce behavioral changes in the population leading to a reduction in the horizontal transmission rate of both HBV and HDV.

We employed two best treatment options currently available for mono-infected and co-infected individuals, PEGylated-Interferon (PEG-IFN) and Tenofovir (TDF). The 48 weeks of PEG-IFN treatment costs approximately 9 times than one-year treatment with TDF; however the efficacy of the PEG-IFN treatment for mono-infected is approximately three times than of TDF. Moreover, the efficacy of TDF for co-infected individuals is zero while PEG-IFN for co-infected is as effective as for mono-infected individuals. The efficacy and costs of all interventions are given in Table 1.

Furthermore, the feasibility of the implementation of an upcoming promising therapy (i.e. 1-year of nucleic acid polymer therapy or NAP) [53, 54] for both mono-infections and co-infections in Brazilian public health system (at the national level) is also tested in a crude manner by assuming different introductory prices. The expected sustained virological response (SVR) efficacy of NAP is 80% for mono-infections and 42% for co-infections [53].

We propose five strategies based on different combinations of five interventions as following,

1. Strategy-1: None of the five interventions.
2. Strategy-2: All five interventions but with no testing for HDV and thus, assuming all infections are mono-infections and the choice of treatment here is 1-year TDF.
3. Strategy-3: All five interventions with testing for HDV and the choice of treatment here is 1-year TDF and 1-year PEG-IFN for mono-infections and co-infections, respectively.
4. Strategy-4: All five interventions with testing for HDV and the choice of treatment here is 4-year TDF and 1-year PEG-IFN for mono-infections and co-infections, respectively.
5. Strategy-5: All five interventions with testing for HDV and the choice of treatment here is 1-year PEG-IFN for all infections.

### Optimal controls

The objective is to simultaneously reduce the number of HBV and HDV infections residing in the population as well as the disease-induced deaths. We employed Genetic Algorithm [55] in MATLAB R2016b to determine the optimal level of the implementation of interventions for a given year under any strategy for a constrained yearly budget (the derivation of costs is given in the section B in S1 File [25]) that is assumed to increase at 11% yearly while the cost of all interventions are discounted at 3% yearly (see section C in S1 File). We also considered an unlimited resources situation to determine the maximum improvement in controlling HBV and HDV epidemics that one can observe on different levels in Brazil, (i) national level (Brazil), (ii) state level (State of Acre), (iii) city level (Manaus and Eirunepé city), and (iv) municipality level (Lábrea Municipality). The prevalence information in all these regions is given in Table 2.

## Results

### Intervention effectiveness with unlimited treatment resources

**Fig 2:**
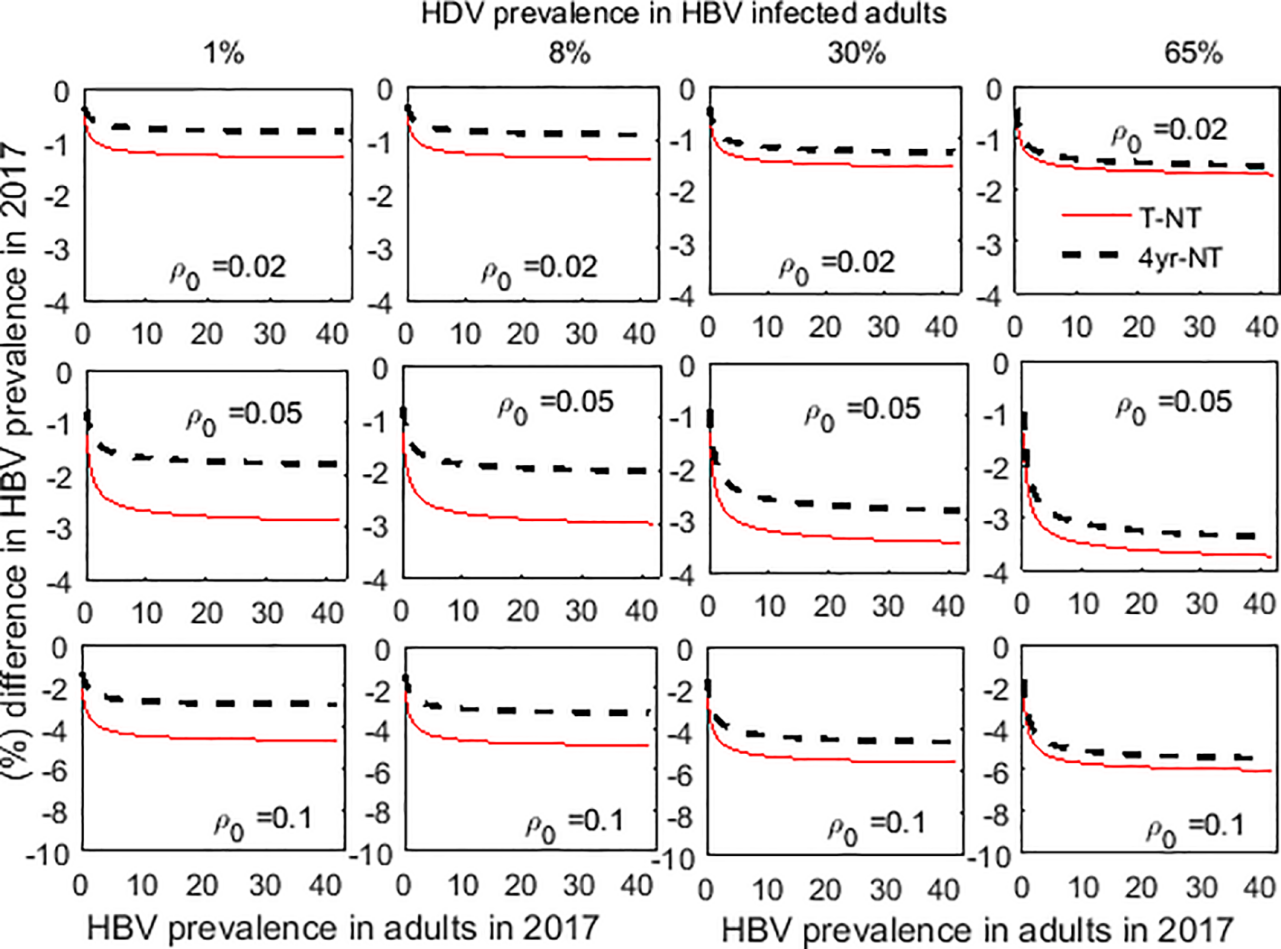
Impact of HDV testing on HBV prevalence according to initial HBV prevalence in adults, HDV prevalence in HBV mono-infected adults and allowed maximum screening rate of the total population. The maximum screening rate (*ρ*_0_) and HDV prevalence in HBV mono-infected individuals are varied across columns and rows, respectively while the prevalence of HBV in the population is varied on the x-axis of each subfigure. The screening rate can be interpreted as approximately the proportion of the population that can be screened each year. The black dashed line shows the reduction in hepatitis prevalence (both mono- and co-infections) from treating HBV-only with a 4-year course of TDF and co-infections with 1-year PEG-IFN, while the red line shows the effect of treating everyone with PEG-IFN.

The effect of interventions on the prevalence of hepatitis is first determined under the assumption that the only limiting factor is the public health infrastructure available to screen people (Fig 2). That is, all infected individuals that are diagnosed through screening are treated, regardless of the cost. This also establishes an upper limit for intervention efficacy for a given level of screening capacity.

When treatment resources are unconstrained, the major determinant of the effect of interventions is the screening rate. Likewise, treating everyone with PEG-IFN is always better regardless of the joint prevalence or the screening rate. This is evident by the fact that the red line representing prevalence with universal PEG-IFN treatment is below the dashed black line prevalence under universal TDF treatment for all plots in Fig 2. In all settings, the effectiveness of interventions implemented also increases as the prevalence of HDV increases. When treatment resources are unconstrained, all co-infected individuals are being treated with PEG-IFN rather than TDF, the latter being ineffective for co-infected individuals. This increased effectiveness comes at a very high cost, as unlimited treatment in high-prevalence settings requires substantial ongoing investment that far surpasses most public health budgets (Figure A in S1 File). However, by assuming that an infection prevented/cured saves at least 10 life years with a quality of life measure of 0.75 [25], we still find that it is cost-effective to explicitly conduct HDV testing and treat HDV infections with PEG-IFN (as the cost per quality-adjusted-life-years (QALYs) saved is always smaller than the 3×GDP ($25,947) of Brazil [56]) (Figure B in S1 File).

### Intervention effectiveness with realistic budgets

**Fig 3:**
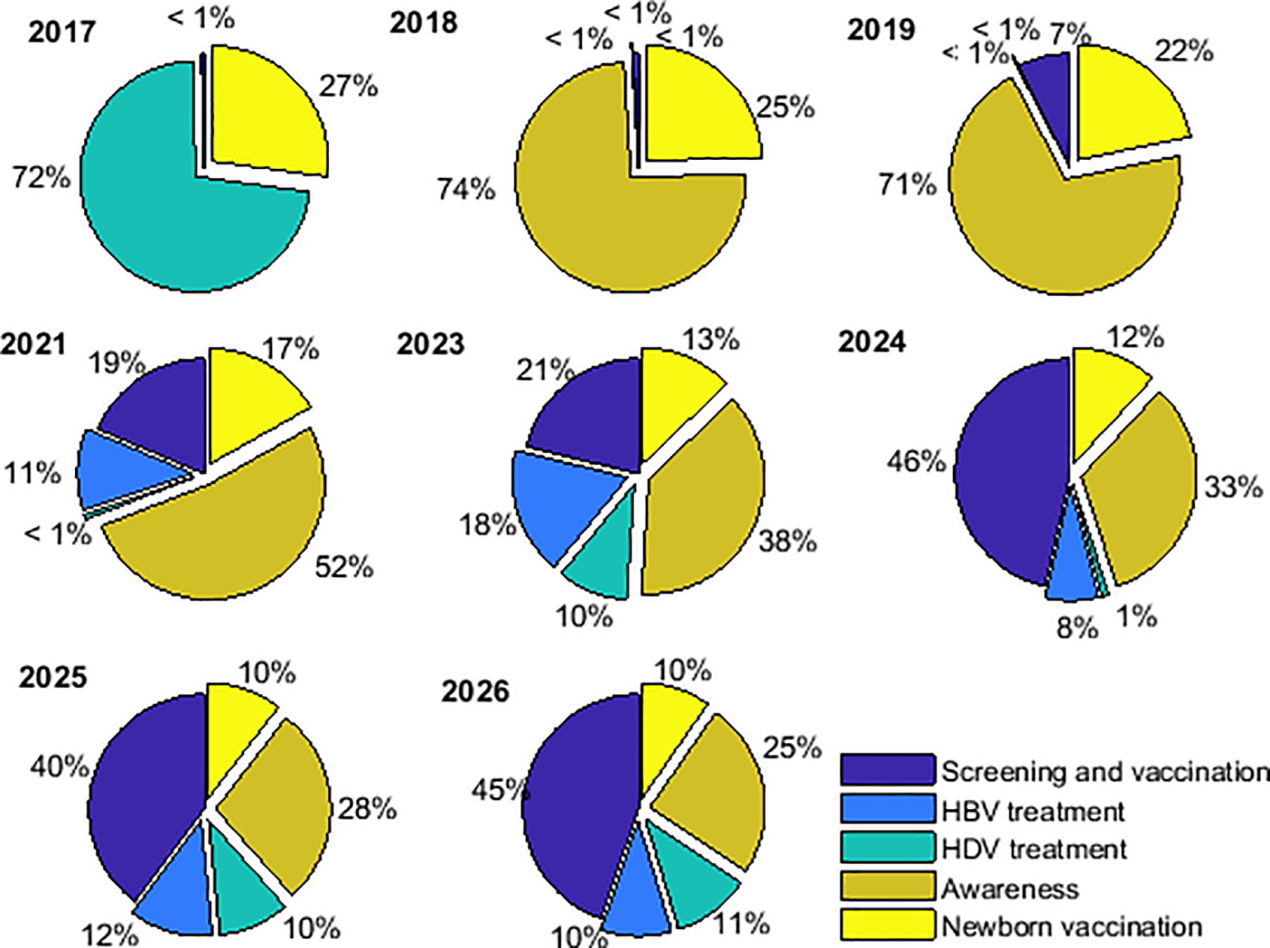
Illustration of the dynamic allocation of the budget among five interventions under strategy 4 in Brazil between 2017 and 2027.

Public health budgets for hepatitis prevention in Brazil are small (less than $1 USD per person-year, see Table 2). In this section, we consider how to optimally allocate resources within 5 intervention strategies at multiple scales in Brazil, which were found to be dynamic on a year to year basis (shown for Brazil under strategy 4 in Fig 3). We attempt to answer 2 questions: 1) does screening for co-infection improve intervention effectiveness, and 2) if so, what is the best treatment strategy for a screening program that tests for both HBV and HDV?

We consider 5 basic scenarios: 1) no intervention to establish a baseline, 2) untargeted intervention that screens for HBV-only (i.e. does not identify co-infection), 3) targeted intervention that treats HBV-only with 1-year TDF, 4) targeted intervention that treats HBV-only with 4-year TDF, 5) targeted intervention that treats HBV-only with 1-year PEG-IFN. In the targeted interventions, all co-infected persons are treated with 1-year PEG-IFN. We also analyze 5 different regions with variable levels of HBV and HDV prevalence: Brazil (Table 3); Manaus, a city in Amazonas state, (Table 4); Lábrea, a municipal region in Amazonas state, (Table 5); Eirunepé, a different city in Amazonas state (Table 6); and, Acre State, a state in Brazil (Table 7).

**Table 3:** Population stats in Brazil in 2027 under five different strategies.

Strategy	*HB*_*P*_ (×10^6^)	*HD*_*HB*_ (×10^3^)	*R*_*P*_ (×10^6^)	*D*_*T*_ (×10^3^)	*C*_*I*_ ($, ×10^6^)	*C*_*R*_ ($, ×10^9^)	*C*_*S*_ ($, ×10^6^)	*C*_*T*_ ($, ×10^6^)
1	1.4908	51.967	5.11	18.241	88.8	5.55	NA	NA
2	1.2948	45.868	31.795	17.265	584.38	4.82	184.3	42.48
3	1.2958	45.012	29.572	17.260	598.36	4.82	170.4	71.68
4	1.2906	45.064	28.813	17.238	594.47	4.80	164.8	71.50
5	1.3015	45.396	23.16	17.265	600.0	4.84	125.7	114.5

Here, we consider 5 strategies: 1) no intervention to establish a baseline, 2) untargeted intervention that screens for HBV-only (i.e. does not identify co-infection), 3) targeted intervention that treats HBV-only with 1-year TDF, 4) targeted intervention that treats HBV-only with 4-year TDF, 5) targeted intervention that treats HBV-only with 1-year PEG-IFN. In the targeted interventions, all co-infected persons are treated with 1-year PEG-IFN.

Stats in 2017: Population: 225 million; the number of HBV infections including mono-infected and dually infected individuals in the population (*HB*_*P*_): 1.54 million; the number of HDV infections in the population (*HD*_*HB*_) = 60,990; recovered population (*R*_*P*_) = 3.04 million; HBV and HDV related death toll over the next 10 years (*D*_*T*_); the combined cost of all interventions over the next 10 years (*C*_*I*_); the cost of residual infections in 2027 (*C*_*R*_); the cost of screening and adult vaccination over the next 10 years (*C*_*S*_); the cost of treatment of HBV and HDV infected individuals over the next 10 years (*C*_*T*_). Here, NA represents not applicable.

**Table 4:** Population stats in Manaus in 2027 under five different strategies.

Strategy	*HB*_*P*_	*HD*_*HB*_	*R*_*P*_	*D*_*T*_	*C*_*I*_ ($, ×10^6^)	*C*_*R*_ ($, ×10^9^)	*C*_*S*_ ($, ×10^6^)	*C*_*T*_ ($, ×10^6^)
1	73,159	10,231	226,160	2028	0.73	0.28	NA	NA
2	65,656	9,896	698,440	2005	9.44	0.24	4.09	1.98
3	64,645	9,531	699,625	1985	20.68	0.24	4.22	13.11
4	64,304	9,481	700,494	1981	20.72	0.24	4.22	13.16
5	63,937	9,477	701,020	1979	20.70	0.24	4.22	13.14

Here, we consider 5 strategies: 1) no intervention to establish a baseline, 2) untargeted intervention that screens for HBV-only (i.e. does not identify co-infection), 3) targeted intervention that treats HBV-only with 1-year TDF, 4) targeted intervention that treats HBV-only with 4-year TDF, 5) targeted intervention that treats HBV-only with 1-year PEG-IFN. In the targeted interventions, all co-infected persons are treated with 1-year PEG-IFN.

Stats in 2017: Population: 2.2 million; the number of HBV infections including mono-infected and dually infected individuals (*HB*_*P*_): 73,287; the number of HDV infections in the population (*HD*_*HB*_): 11,955; recovered population (*R*_*P*_): 96,407; HBV and HDV related death toll over the next 10 years (*D*_*T*_); the combined cost of all interventions over the next 10 years (*C*_*I*_); the cost of residual infections in 2027 (*C*_*R*_); the cost of screening and adult vaccination over the next 10 years (*C*_*S*_); the cost of treatment of HBV and HDV infected individuals over the next 10 years (*C*_*T*_). Here, NA represents not applicable.

**Table 5:** Population stats in Lábrea municipality in 2027 under five different strategies.

Strategy	*HB*_*P*_	*HD*_*HB*_	*R*_*P*_	*D*_*T*_	*C*_*I*_ ($, ×10^4^)	*C*_*R*_ ($, ×10^6^)	*C*_*S*_ ($, ×10^4^)	*C*_*T*_ ($, ×10^4^)
1	2436	213	7165	53	1.49	9.06	NA	NA
2	2128	203	15220	52	42.19	7.92	7.92	27.39
3	2106	199	15361	52	42.29	7.83	8.48	26.93
4	2093	197	15409	51	42.52	7.79	8.52	27.09
5	2083	197	15403	51	42.37	7.75	8.50	26.95

Here, we consider 5 strategies: 1) no intervention to establish a baseline, 2) untargeted intervention that screens for HBV-only (i.e. does not identify co-infection), 3) targeted intervention that treats HBV-only with 1-year TDF, 4) targeted intervention that treats HBV-only with 4-year TDF, 5) targeted intervention that treats HBV-only with 1-year PEG-IFN. In the targeted interventions, all co-infected persons are treated with 1-year PEG-IFN.

Stats in 2017: Population: 45,306; the number of HBV infections including mono-infected and dually infected individuals (*HB*_*P*_): 2422; the number of HDV infections in the population (*HD*_*HB*_): 246; recovered population (*R*_*P*_): 3088; HBV and HDV related death toll over the next 10 years (*D*_*T*_); the combined cost of all interventions over the next 10 years (*C*_*I*_); the cost of residual infections in 2027 (*C*_*R*_); the cost of screening and adult vaccination over the next 10 years (*C*_*S*_); the cost of treatment of HBV and HDV infected individuals over the next 10 years (*C*_*T*_). Here, NA represents not applicable.

**Table 6:** Population stats in Eirunepé city in 2027 under five different strategies.

Strategy	*HB*_*P*_	*HD*_*HB*_	*R*_*P*_	*D*_*T*_	*C*_*I*_ ($, ×10^4^)	*C*_*R*_ ($, ×10^6^)	*C*_*S*_ ($, ×10^4^)	*C*_*T*_ ($, ×10^4^)
1	995	210	2947	36	1.16	3.70	NA	NA
2	867	201	10822	35	14.53	3.27	6.58	2.58
3	859	194	10686	35	32.82	3.20	6.57	20.97
4	854	193	10743	35	32.92	3.18	6.62	20.97
5	848	193	10660	35	32.95	3.16	6.56	21.03

Here, we consider 5 strategies: 1) no intervention to establish a baseline, 2) untargeted intervention that screens for HBV-only (i.e. does not identify co-infection), 3) targeted intervention that treats HBV-only with 1-year TDF, 4) targeted intervention that treats HBV-only with 4-year TDF, 5) targeted intervention that treats HBV-only with 1-year PEG-IFN. In the targeted interventions, all co-infected persons are treated with 1-year PEG-IFN.

Stats in 2017: Population: 35,237; the number of HBV infections including mono-infected and dually infected individuals (*HB*_*P*_): 973; the number of HDV infections in the population (*HD*_*HB*_): 246; recovered population (*R*_*P*_): 1252; HBV and HDV related death toll over the next 10 years (*D*_*T*_); the combined cost of all interventions over the next 10 years (*C*_*I*_); the cost of residual infections in 2027 (*C*_*R*_); the cost of screening and adult vaccination over the next 10 years (*C*_*S*_); the cost of treatment of HBV and HDV infected individuals over the next 10 years (*C*_*T*_). Here, NA represents not applicable.

**Table 7:** Population stats in Acre state in 2027 under five different strategies.

Strategy	*HB*_*P*_	*HD*_*HB*_	*R*_*P*_	*D*_*T*_	*C*_*I*_ ($, ×10^6^)	*C*_*R*_ ($, ×10^6^)	*C*_*S*_ ($, ×10^6^)	*C*_*T*_ ($, ×10^6^)
1	11,156	2,529	32,979	395	0.27	41.50	NA	NA
2	9,805	2,484	241,335	395	3.12	36.48	1.60	0.25
3	9,710	2,391	241,432	390	3.76	36.13	1.61	0.88
4	9,716	2,391	241,433	390	3.81	36.15	1.61	0.93
5	9,585	2,370	241,458	390	7.79	35.66	1.61	4.93

Here, we consider 5 strategies: 1) no intervention to establish a baseline, 2) untargeted intervention that screens for HBV-only (i.e. does not identify co-infection), 3) targeted intervention that treats HBV-only with 1-year TDF, 4) targeted intervention that treats HBV-only with 4-year TDF, 5) targeted intervention that treats HBV-only with 1-year PEG-IFN. In the targeted interventions, all co-infected persons are treated with 1-year PEG-IFN.

Stats in 2017: Population: 835,670; the number of HBV infections including mono-infected and dually infected individuals (*HB*_*P*_): 10,743; the number of HDV infections in the population (*HD*_*HB*_): 2,977; recovered population (*R*_*P*_): 15909; HBV and HDV related death toll over the next 10 years (*D*_*T*_); the combined cost of all interventions over the next 10 years (*C*_*I*_); the cost of residual infections in 2027 (*C*_*R*_); the cost of screening and adult vaccination over the next 10 years (*C*_*S*_); the cost of treatment of HBV and HDV infected individuals over the next 10 years (*C*_*T*_). Here, NA represents not applicable.

Comparing the no-intervention strategy (baseline) to any other intervention strategy, we see that intervention effects are modest, which is expected given the low-efficacy of treatment and limited treatment resources. However, we also see that even with these highly limited budgets, it is possible to see at least 189,300 fewer cases under strategy 5 at the national level compared to the baseline (calculated as *HB*_*P*_ under strategy 5 minus *HB*_*P*_ under strategy 1 in Table 3).

To address the first posed question, we compared strategy 2 to strategies 3, 4, and 5. Strategy 2 (the untargeted strategy) was never the best option. This result makes it clear that the additional cost of screening and the use of a more expensive PEG-IFN are offset by not wasting resources on treating co-infected persons with TDF. The difference between strategy 2 and strategies 3, 4, and 5 are the least pronounced at the national level (Table 3). This is because the national prevalence of HBV is much lower (~0.6%) than in specific sub-regions. Areas with high prevalence of co-infection will generally benefit less from targeted screening; however, even in low HBV and moderate HDV endemic region, we see that some of the targeted screenings outperform the untargeted strategy.

In all sub-national regions, we see that strategy 5 is always the best in terms of reducing the joint prevalence of HBV and HDV along with hepatitis caused mortality. However, the differences between treating HBV mono-infected individuals with either 4 years of TDF or PEG-IFN were very small suggesting that either strategy may be effective in smaller regions. Generally, in high HDV prevalence regions of Brazil, we can say that the increased cost of treating everyone with PEG-IFN is offset by better public health outcomes even with small budgets. Likewise, if PEG-IFN is not available, it is better to treat with 4 years of TDF than 1 year. However, the efficacy of treating everyone with PEG-IFN declines when the prevalence of HDV is low.

We also performed a conservative cost-effectiveness analysis at the national level by assuming that an infection prevented/cured saves at least 10 life years with a quality of life measure of 0.75 [25]. In general, all of the hypothetical interventions are cost-effective; yet different interventions have different population-level effects even when budgets are small. With the most number of infections prevented/cured, the strategy 4 would save at least 1.42×10^6^ quality-adjusted-life-years (QALYs) (calculated as the assumed number of number of quality life years saved per prevented case (10 years * 0.75 = 7.5 years) times the number of prevented cases (*HB*_*P*_ under strategy 4 minus *HB*_*P*_ under strategy 1 in Table 3)) but at an additional cost of $511 million compared to the baseline, which yields cost per QALY as $388, which is significantly higher than the 3×GDP ($25,947) of Brazil [56].

Furthermore, a doubling of the budget leads to an additional 27,600 infections being prevented/recovered at the national level while at the regional level, the impact of budget doubling was either modest or insignificant (Table 8).

**Table 8:** Impact of budget doubling in Brazil at the national level and regional level on the number of HBV and HDV infections in 2027.

Region	Strategy	*HB*_*P*_ when budget is doubled	*HB*_*P*_ when budget is at its current value	Decrease in *HB*_*P*_ due to budget doubling
Brazil	4-yr TNF for mono-infections 1-yr PEG-IFN for co-infections	1.2630 ×10^6^	1.2906×10^6^	27600
Acre State	1-yr PEG-IFN for mono- and co-infections	9508	9585	78
Eirunepé	1-yr PEG-IFN for mono- and co-infections	845	848	3
Lábrea	1-yr PEG-IFN for mono- and co-infections	2082	2083	12
Manaus	1-yr PEG-IFN for mono- and co-infections	63710	63937	227

In these strategies, awareness programs, screening, newborn and adult HBV vaccination is included along with the treatment mentioned in the Table

The number of HBV infections including mono-infected and dually infected individuals is given by *HB*_*P*_.

### Will the implementation of upcoming NAP therapy be better than existing options and at what introductory price?

**Fig 4:**
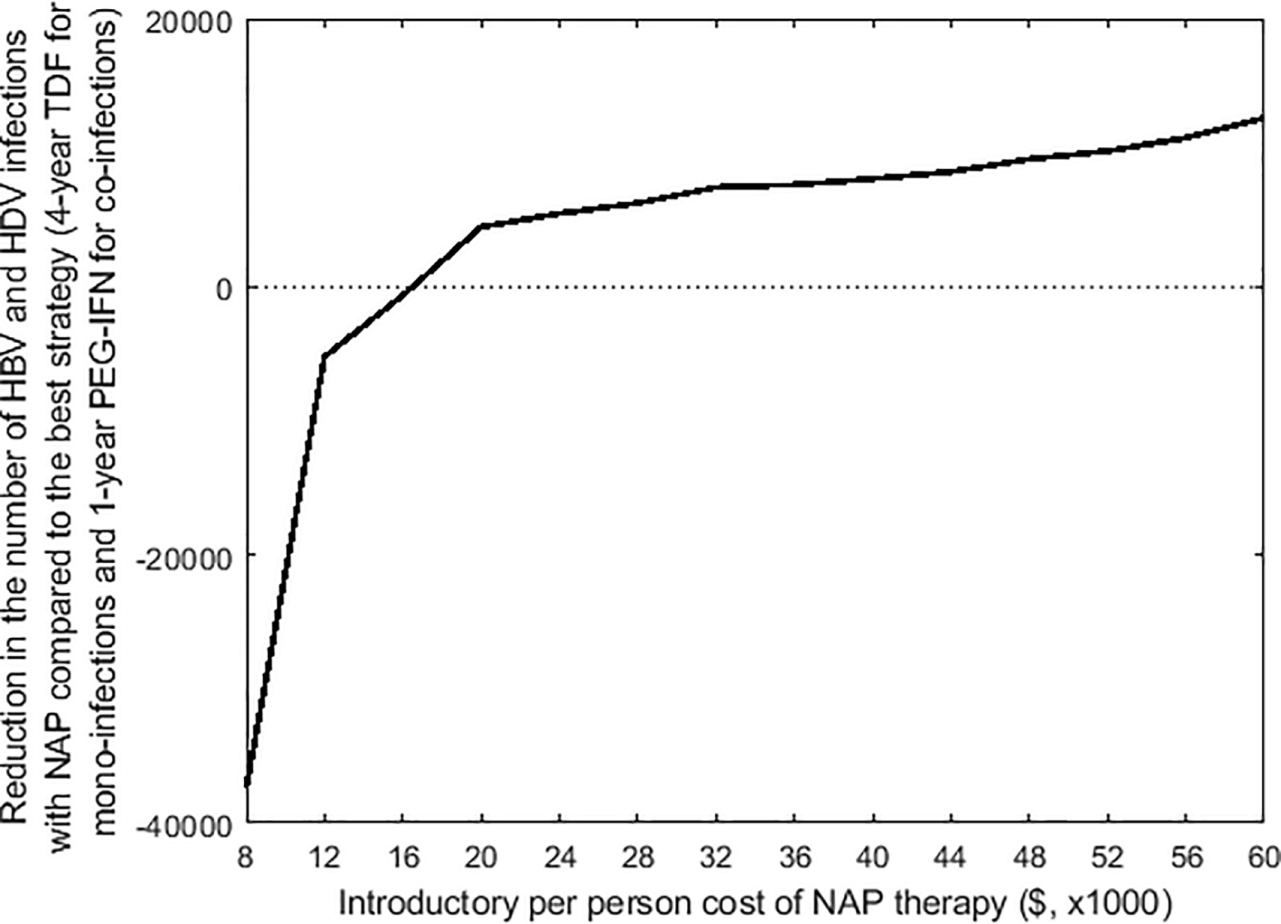
Comparison of the number of cases averted 2017–2027 for universal 1-year NAP therapy to 1-year PEG-IFN for co-infections and 4-year TDF for mono-infections. The x-axis gives the cost per course of NAP treatment and the y-axis show the number of additional cases caused by using universal NAP therapy instead of alternative 1-year PEG-IFN for co-infections and 4-year TDF for mono-infections (negative values indicated prevented cases). Universal NAP therapy compared to PEG-IFN for co-infections and TDF for mono-infections are approximately equal at a cost of about $16,000 for a single course of NAP.

One upcoming promising therapy for both mono-infections and coinfections is NAP [53, 54], which might be available soon. With an expected introductory price of ~$50,000 (assuming similar to PEG-IFN, when it was first introduced), we found that despite high efficacy of NAP treatment, its inclusion will have less impact in reducing the joint prevalence of two viruses than the existing best treatment option in Brazil (4-year TDF for mono-infections and 1-year Peg-IFN for co-infections) (Fig 4). The introductory price of NAP treatment must be less than $16,000 per person to become competitive with existing therapies.

## Discussion

In Brazil, mono-infections and co-infections are generally isolated to regions with the lowest access to health services and lowest prevention budgets. Moreover, the natural barriers that keep HDV contained in certain regions of Brazil are breaking down with increasing industrialization and access to remote areas. Given the high levels of HBV infection in Brazil and the surrounding countries, control and containment of HDV is essential and increasingly important for the broader population health of the entire region and potentially the entire world. Although, the Brazilian government created the National Viral Hepatitis Program in 2012, optimization of the available resources to contain/eradicate HBV and HDV epidemics is missing.

This model suggests that, given realistic budgets and heterogeneous levels of both HBV and HDV infection in various regions of Brazil, screening for HDV provides additional population-level benefits that offset the additional costs. Moreover, WHO does not recommend PEG-IFN or IFN but only Entecavir/Tenofovir due to cost issues in developing regions with limited resources [30], while also not factoring HDV in their analysis. However, our analysis supports that PEG-IFN, though more expensive, generally produces greater reductions in the incidence and prevalence of HBV in the general population, in consistent with [57]. Our analysis also agrees with the suggested implementation of free HBV and HDV screening followed by an adequate treatment and vaccination strategy in China, another resource-constrained environment [58]. Our model also allowed us to make an estimate of the cost per QALY saved ($388) for the best treatment strategy (4 year TDF for mono-infections and 1 year PEG-IFN for co-infections), which was in line with previous estimates for universal TDF treatment [41]. Despite low efficacy, a substantial investment was also made to awareness programs as they played an important role in the optimal control of HDV and HBV in Brazil [59].

Moreover, one of the significant barriers to HBV (and HDV) control is the lack of effective and inexpensive therapies. New therapies such as NAP that may become available soon have shown promising results in clinical trials [53, 54], but the expected high cost of new pharmaceuticals may render the treatment useless on a population level. We expect that only when the introductory price of NAP would be less than $16,000, it would be beneficial for the Brazilian government to include it in public health system over currently available therapeutic options.

Despite the usefulness of our model, it can be further improved. For example, future models can reflect that TDF receiving mothers have a very low probability of vertical transmission [60]. Furthermore, one can also incorporate the treatment for HBV-infected children [61, 62], with SVR of 10% under 24 weeks IFN therapy and SVR of 2% under 72 weeks of TDF treatment [63]. Unfortunately, there are no pediatric recommendations to treat HDV currently [64]. The fact that we do not know the additional amount spent at the state level by state governments on hepatitis viruses means that our state-level budgets are likely underestimates [45]. However, our results suggest that small changes to the total budget will not change the inference qualitatively and the quantitative effect will be a small underestimation of the number of preventable cases.

In conclusion, we support an additional screening for HDV even in a low HBV and HDV endemic region. Moreover, we recommend PEG-IFN, wherever possible, for all infections. However, if PEG-IFN is not available in abundance, PEG-IFN for co-infections and 4-year TDF treatment for mono-infections is highly desirable to achieve HBV and HDV control, even in countries where the per capita health budget is less than $1.

## Supporting information

S1 File Supplementary material and methods. Detailed explanation and formulation of the mathematical model, cost functions, parameter values used in the model.
(DOCX)
