# Supplementary material for: Screening for hepatitis D and PEG-Interferon over Tenofovir enhance general hepatitis control efforts in Brazil
Source: PLoS One. 2018 Sep 7;13(9):e0203831. doi: 10.1371/journal.pone.0203831 (PMC6128631; doi:10.1371/journal.pone.0203831)
Supplement: S1 File — Detailed explanation and formulation of the mathematical model, cost functions, parameter values used in the model. (DOCX) [file pone.0203831.s001.docx]

S1 File: Supplementary Material and Methods: Detailed explanation of the mathematical model, cost functions, parameter values used in the model.

## **Section A: Mathematical model**

In the model,$X$ and $Y$ represent susceptible and infected population class respectively while $R$ represents recovered population class. Mono-HBV infected individuals and HBV-HDV co-infected individuals are represented by subscripts $b$ and $bd$ respectively. Subscripts$1$, $2$, $u$, $F$ and $T$ denote acute phase, chronic phase, untested individuals, individuals who failed treatment and individuals under treatment, respectively. A person is assumed to be in acute phase upon diagnosis with HBV and the persistence of HBV viremia for more than 6 consecutive months is identified as chronic phase [1]. Borrowed from the ref [1], the different children compartments are: $R_{u}$ vaccinated; $X_{u}$ unvaccinated and uninfected and $Y_{ub}$ unvaccinated and HBV infected. Similarly, the different adult compartments are: $X$ susceptible; $R_{v}$ vaccinated in the childhood;$Y_{a2u}$ undiagnosed chronic HBV infections originated from unvaccinated and infected children; $Y_{b1u}$ undiagnosed acute HBV infections; $Y_{b2u}$ undiagnosed chronic HBV infections; $Y_{b1}$ diagnosed acute HBV infections; $Y_{b2}$ diagnosed chronic HBV infections; $Y_{bd1u}$ undiagnosed acute HBV-HDV co-infections; $Y_{bd2u}$ undiagnosed chronic HBV-HDV co-infections; $Y_{bd1}$ diagnosed acute HBV-HDV co-infections; $Y_{bd2}$ diagnosed chronic HBV-HDV co-infections; $Y_{bF}$ HBV mono-infected infections who failed treatment; $Y_{bdF}$ HBV-HDV dual infections who failed treatment; $R$ adults who recovered from mono-infection or coinfection naturally; $R_{R}$ recovered who status as recovered is also known; $Y_{bT1}$ HBV mono-infections who failed one year of Tenofovir treatment; $Y_{bT2}$ HBV mono-infections who failed two consecutive years of Tenofovir treatment; $Y_{bT3}$ HBV mono-infections who failed three consecutive years of Tenofovir treatment. In addition to the compartments in the ref [1], we also included one more compartment which children infected with HDV ($Y_{ud}$) through household transmission. Our model is also inclusive of following five interventions,

(i) $\rho_{1}(t)$: the probability of diagnosed mono-infected adults receiving antiviral treatment at time $t$ with the corresponding rate $u_{1}\left( t \right)$ derived from the relation $\rho_{1}\left( t \right)=1-e^{-u_{1}(t)}$ in the model,

(ii)$\rho_{2}(t)$: the probability of diagnosed dually-infected adults receiving antiviral treatment at time $t$ with the corresponding rate $u_{2}\left( t \right)$ derived from the relation $\rho_{2}\left( t \right)=1-e^{-u_{2}(t)}$ in the model,

(iii) $u_{3}(t)$: the intensity of awareness programs at time $t$. This intervention aims at promoting safer sex in the total population to reduce horizontal transmission of HBV and HDV infection [2]. Here, we assume $\rho_{3}\left( t \right)=u_{3}(t)$.

(iv) $\rho_{4}(t)$: the probability of testing and vaccinating of an individual in the eligible population and with the corresponding rate $u_{4}\left( t \right)$ derived from the relation $\rho_{4}\left( t \right)=\frac{1}{\rho_{0}}(1-e^{-u_{4}\left( t \right)})$ in the model. Here the parameter $\rho_{0}$ describes the fraction of the adult population that can be tested at time $t$. A person is initially tested for HBV and then for HDV [3] but only when an adult is HBV positive. On the other hand, if a person is categorized as a susceptible, then adult vaccination is administered in that person.

(v) $u_{5}(t)$: an increase in the intensity of new-born vaccination coverage from the current coverage $\beta$. Overall, HBV new-born vaccination coverage at time $t$ is given by $\Omega\left( t \right)=\beta+(1-\beta)u_{5}\left( t \right)$. Here, we assume $\rho_{5}\left( t \right)=u_{5}(t)$.

In the modelling process, we further assume that those individuals who receive treatment do not contribute in the spread of HBV and HDV infection as they become aware of their infection status [1]. The model is given by,

$\frac{{dR}_{u}}{dt}=\mu_{r}\omega_{c}\Omega\left( t \right)(N-R_{u}-X_{u}-Y_{u}-Y_{ud})-\delta_{X}R_{u}-\alpha R_{u}-\mu R_{u}$ (1)

$\frac{dX_{u}}{dt}=\mu_{r}\left( 1-\Omega\left( t \right) \right)\left( X+R_{v}+R+R_{R} \right)-\delta_{X}X_{u}-\alpha X_{u}-\mu X_{u}-\psi X_{u}\left( Y_{bd1u}+Y_{bd2u}+Y_{bd1}+Y_{bd2}+Y_{bdF} \right)-\psi X_{u}\left( Y_{ub}+Y_{b1u}+Y_{b2u}+Y_{b1}+Y_{b2}+Y_{bd1u}+Y_{bd2u}+Y_{bd1}+Y_{bd2}+Y_{bF}+Y_{bdF} \right)$ (2)

$\frac{dY_{ub}}{dt}=\mu_{r}\left( 1-\Omega\left( t \right) \right)\left( N-\left( {R_{u}+X}_{u}+Y_{ub}+Y_{ud}+X+R_{v}+R+R_{R} \right) \right)+\psi X_{u}\left( Y_{ub}+Y_{b1u}+Y_{b2u}+Y_{b1}+Y_{b2}+Y_{bd1u}+Y_{bd2u}+Y_{bd1}+Y_{bd2}+Y_{bF}+Y_{bdF} \right)-\psi Y_{ub}\left( Y_{ud}+Y_{bd1u}+Y_{bd2u}+Y_{bd1}+Y_{bd2}+Y_{bdF} \right)-\left( \delta_{X}+\alpha+\mu\right)Y_{ub}$ (3)

$\frac{dY_{ud}}{dt}=\psi X_{u}\left( Y_{bd1u}+Y_{bd2u}+Y_{bd1}+Y_{bd2}+Y_{bdF} \right)+\psi Y_{ub}(Y_{ud}+Y_{bd1u}+Y_{bd2u}+Y_{bd1}+Y_{bd2}+Y_{bdF})-(\delta_{X}+\alpha+\mu)Y_{ud}$ (4)

$\frac{dX}{dt}=\delta_{X}X_{u}-X\left( G_{b}+F_{b}+F_{bd} \right)-\mu X-u_{4}\left( t \right)\omega_{a}X$ (5)

${\frac{dR_{v}}{dt}=\delta}_{X}R_{u}-\mu R_{v}$ (6)

$\frac{dY_{a2u}}{dt}=0.95\delta_{X}Y_{ub}-Y_{a2u}F_{d}-(\mu+u_{4}\left( t \right)+\theta_{b2}+\nu_{b2})Y_{a2u}$ (7)

$\frac{dY_{b1u}}{dt}=X\left( G_{b}+F_{b} \right)-Y_{b1u}F_{d}-(\mu+u_{4}\left( t \right)+\theta_{b1}+\gamma_{b})Y_{b1u}$ (8)

$\frac{dY_{b2u}}{dt}=\gamma_{b}Y_{b1u}-Y_{b2u}F_{d}-(\mu+u_{4}\left( t \right)+\theta_{b2})Y_{b2u}-\nu_{b2}Y_{b2u}$ (9)

$\frac{dY_{b1}}{dt}=u_{4}\left( t \right)Y_{b1u}-Y_{b1}F_{d}-\left( \mu+\theta_{b1}+\gamma_{b} \right)Y_{b1}$ (10)

$\frac{dY_{b2}}{dt}=u_{4}\left( t \right)(Y_{b2u}+Y_{a2u})+\gamma_{b}Y_{b1}-Y_{b2}F_{d}-\left( \mu+\theta_{b2} \right)Y_{b2}-\nu_{b2}Y_{b2}-u_{1}\left( t \right)Y_{b2}$ (11)

$\frac{dY_{bd1u}}{dt}=XF_{bd}+\left( Y_{a2u}+Y_{b1u}+Y_{b2u}+Y_{b1}+Y_{b2}+Y_{bF} \right)F_{d}-(\mu+u_{4}\left( t \right)+\theta_{bd1}+\gamma_{bd})Y_{bd1u}$ (12)

$\frac{dY_{bd2u}}{dt}=\delta_{X}Y_{ud}+\gamma_{bd}Y_{bd1u}-(\mu+u_{4}\left( t \right)+\theta_{bd2})Y_{bd2u}-\nu_{bd2}Y_{bd2u}$ (13)

$\frac{dY_{bd1}}{dt}=u_{4}\left( t \right)Y_{bd1u}-\left( \mu+\theta_{bd1}+\gamma_{bd} \right)Y_{bd1}-u_{2}\left( t \right)Y_{bd1}$ (14)

$\frac{dY_{bd2}}{dt}=u_{4}\left( t \right)Y_{bd2u}+\gamma_{bd}Y_{bd1}-\left( \mu+\theta_{bd2} \right)Y_{bd2}-\nu_{bd2}Y_{bd2}-u_{2}\left( t \right)Y_{bd2}$ (15)

$\frac{dY_{bF}}{dt}={(1-\epsilon}_{14})100Y_{bT3}-Y_{bF}F_{d}-\mu Y_{bF}-\nu_{b2}Y_{bF}$ (16)

$\frac{dY_{bdF}}{dt}={(1-\epsilon}_{2})u_{2}\left( t \right){(Y}_{bd1}+Y_{bd2})-\mu Y_{bdF}-\nu_{bd2}Y_{bdF}$ (17)

$\frac{dR}{dt}=0.05\delta_{X}Y_{ub}+\theta_{b1}\left( Y_{b1}+Y_{b1u} \right)+\theta_{b2}{(Y}_{b2}+Y_{b2u}+Y_{a2u})+\theta_{bd1}{(Y}_{bd1}+Y_{bd1u})+\theta_{bd2}(Y_{bd2}+Y_{bd2u})-u_{4}\left( t \right)R-\mu R$ (18)

$\frac{dR_{R}}{dt}={u_{1}\left( t \right)\epsilon}_{1}Y_{b2}+100(\epsilon_{12}Y_{bT1} +\epsilon_{13}Y_{bT2}+\epsilon_{14}Y_{bT3})+u_{2}\left( t \right)\epsilon_{2}{(Y}_{bd1}+Y_{bd2} )+u_{4}\left( t \right)\omega_{a}X+u_{4}\left( t \right)R-\mu R_{R}$ (19)

$\frac{dY_{bT1}}{dt}={(1-\epsilon}_{1})u_{1}\left( t \right)Y_{b2}-\mu Y_{bT1} -100Y_{bT1}$ (20)

$\frac{dY_{bT2}}{dt}={(1-\epsilon}_{12}){100Y}_{bT1}-\mu Y_{bT2} -100Y_{bT2}$ (21)

$\frac{dY_{bT3}}{dt}={(1-\epsilon}_{13}){100Y}_{bT2}-\mu Y_{bT3} -100Y_{bT3}$ (22)

where,

$G_{b}=\phi\left( 1-\epsilon_{3}u_{3}\left( t \right) \right)\left( \sum_{j=1,2} (p_{bj}Y_{bju}+\nu p_{bj}Y_{bj} \right)+p_{b2}Y_{a2u}+\nu p_{b2}Y_{bF})/N$

$F_{b}=\phi\left( 1-\epsilon_{3}u_{3}\left( t \right) \right)(\sum_{j=1,2} {(q}_{bj}\left( 1-q_{dj} \right)Y_{bdju}+\nu q_{bj}\left( 1-\nu q_{dj} \right)Y_{bdj})+\nu q_{b2}(1-\nu q_{d2})Y_{bdF})/N$

$F_{d}=\phi\left( 1-\epsilon_{3}u_{3}\left( t \right) \right)(\sum_{j=1,2} {(q}_{dj}Y_{bdju}+{\nu q}_{dj}Y_{bdj})+\nu q_{d2}Y_{bdF})/N$

$F_{bd}=\phi\left( 1-\epsilon_{3}u_{3}\left( t \right) \right)(\sum_{j=1,2} (q_{bj}q_{dj}Y_{bdju}+\nu^{2}q_{bj}q_{dj}Y_{bdj})+\nu^{2}q_{b2}q_{d2}Y_{bdF})/N$

Here,

$N={R_{u}+X_{u}+Y_{ub}+Y_{ud}+R}_{v}+Y_{a2u}+X+Y_{b1u}+Y_{b2u}+Y_{b1}+Y_{b2}+Y_{bd1u}+Y_{bd2u}+Y_{bd1}+Y_{bd2}+Y_{bF}+Y_{bdF}+R+R_{R}+Y_{bT1}+Y_{bT2}+Y_{bT3}$.

A detailed description of all model parameters representative is provided in S1 Table.

Table A: Parameter description and their values. All costs are in US dollars.

| Parameter | Description | Value | Reference |
| --- | --- | --- | --- |
| $\mu_{r}$ | Birth rate | 0.015/year | [4] |
| $\mu$ | Death rate | 0.006/year | [4] |
| $\omega_{c}$ | Efficacy of newborn HBV vaccination | 95% | [5-7] |
| $\omega_{a}$ | Efficacy of adult HBV vaccination | 95% | [8] |
| $\phi$ | Rate of partner change | 1.32 partners/year ^a^ |  |
| $\gamma_{b}$ | Rate of progression from acute to chronic HBV infection | 0.4/person/year | [9, 10] |
| $\beta$ | HBV vaccination coverage in new-borns | 96% (Brazil)  80% (Amazon Basin) | [11, 12] |
| $\theta_{b1}$ | Rate of adult recovery from acute HBV infection | 3.6/person/year | [10, 13] |
| $\theta_{b2}$ | Rate of adult recovery from chronic HBV infection | 0.02/person/year | [10, 13] |
| $p_{b1}$ | Probability of HBV transmission from an individual in the HBV acute phase | 0.46 | [10, 13] |
| $p_{b2}$ | Probability of HBV transmission from an individual in the HBV chronic phase | 0.65$p_{b1}$ $p_{b1}$ | [10, 13] |
| $\gamma_{bd}$ | Rate of progression from acute to chronic in dually infected | 2/person/year | [10, 13] |
| $\theta_{bd1}$ | Rate of recovery from acute dual infection | 2/person/year | [10, 13] |
| $\theta_{bd2}$ | Rate of recovery from chronic dual infection | 0.02/person/year | [10, 13] |
| $q_{bj}$ | Probability of HBV transmission from dually infected individual at stage j=1,2 | 0.71$p_{bj}$ | [10, 13] |
| $q_{dj}$ | Probability of HDV transmission from dually infected individual at stage j=1,2 | $q_{bj}$ | [10, 13] |
| $\delta_{X}$ | Rate of maturation of children to adulthood | 1/14 /year | [14] |
| $\nu_{b2}$ | Disease induced mortality rate in chronically HBV infected individuals | 0.0013 /year | [9, 13] |
| $\nu_{bd2}$ | Disease induced mortality rate in chronically dually infected individuals | 0.013 /year | [9, 13] |
| $\alpha$ | Extra-mortality rate during childhood | 0.01 /year | [1] |
| $W_{1}$ | 3-dose new-born HBV vaccination cost | $3.77/person ^b^ | [11, 15] |
| $W_{2}$ | HBV testing cost or HDV testing cost | $3.37/person ^b^ | [16] |
| $W_{3}$ | 3-dose adult HBV vaccination cost | $4.08/person ^b^ | [11, 15] |
| $W_{4}$ | Cost associated with mono-infected individuals treatment | $8172.34/person/year (48 week Peg-Interferon)  $934/person/year (1 year Tenofovir) | [17-19] |
| $W_{5}$ | Cost associated with dually-infected individuals with Peg-Interferon treatment | $8172.34/person/year | [17] |
| $W_{6}$ | Awareness programs cost | $0.2/person/year ^b^ | [9] |
| $\epsilon_{1}$ | Antiviral therapy efficacy for HBV mono-infected individuals | 0.1 ^c^ (48 week Peg-Interferon)  0.032 ^c^ (1 year Tenofovir)  0.075 ^c^ (4 year Tenofovir) | [20-22] |
| $\epsilon_{2}$ | Antiviral therapy efficacy for dually infected individuals with | 0.1 ^c^ (48 week Peg-Interferon)  0 (1 year Tenofovir)  0 (4 year Tenofovir) | [23] |
| $\epsilon_{3}$ | Awareness programs efficacy | 0.5 (Brazil)  0.1 (Amazon Basin) | [9] |
| $T$ | Time interval | 10 years | Assumed |
| $r$ | Annual discount rate | 3% (1-5%) | [9] |
| $\nu$ | Reduction in sexual contact rate after positive diagnosis for HBV and/or HDV | 0.5 (Brazil)  0.1 (Amazon Basin) | [9] |
| $L_{BT}$ | Life time cost of a residual HBV or HDV infection | $5000 ^b^ |  |
| $\psi$ | Household transmission rate | 0 (Brazil) ^d^  SS (Amazon Basin) ^d^ |  |

^a^ Chinese population have the most number of sexual partners in the world, with Brazil stands at the second place [24]. Therefore, the rate of change of sexual partner in Brazil is assumed to be 1.32 which is less than of 1.64 in China [9] but more than 1[25].

^b^ The medical costs in Brazil are approximately 12 times less than the US [11]. Therefore, wherever costs were not available for Brazil, we assumed them to be one-twelfth of the medical costs in the US. The costs reported here are inclusive of both medical and non-medical costs.

^c^ HBsAg seroconversion for mono-infected individuals is being considered as an indicator of sustained virological response [26]. The efficacy with Peg-Interferon treatment also reflects post-treatment efficacy [21]. The year 2 ($\epsilon_{12}$), year 3 ($\epsilon_{13}$) and year 4 ($\epsilon_{14}$) efficacy of Tenofovir therapy was assumed 0.014, 0.014 and 0.014 respectively. For the treatment of HDV coinfected patients, the sustained virological response is reflected by the achievement of HBsAg seroconversion and HDV RNA negativity on treatment and post-treatment, without relapse [23, 27].

^d^ As we do not know nothing about the household size or transmission routes within household transmission or interactions between households in the community [28], we evaluate the household transmission rate by assuming the steady state of the model and using the population size of infected children with HDV in 2017 (or, $Y_{ud}(0)$) and this yields,

$\psi=\frac{(\delta_{X}+\alpha+\mu)Y_{ud}(0)}{X_{u}\left( 0 \right)\left( Y_{bd1u}\left( 0 \right)+Y_{bd2u}\left( 0 \right)+Y_{bd1}\left( 0 \right)+Y_{bd2}\left( 0 \right)+Y_{bdF}\left( 0 \right) \right)+Y_{ub}(0)(Y_{ud}\left( 0 \right)+Y_{bd1u}\left( 0 \right)+Y_{bd2u}\left( 0 \right)+Y_{bd1}\left( 0 \right)+Y_{bd2}\left( 0 \right)+Y_{bdF}\left( 0 \right))}$,

which we assumed true for sub-regions in the Amazon Basin. Since, $Y_{ud}(0)$ is not known at the national level, we excluded household transmission at the national level and thus assumed $\psi=0$.

## **Section B: Costs associated with interventions**

The costs of interventions at a particular time ‘$t_{j}$’ (where $t_{j}$ is year $j$ after the commencement of the program $j=0,\ldots9$) are given by $I_{1}\left( t_{j} \right)$ to $I_{5}(t_{j})$ with a discount rate $r$ [9, 29, 30]:

HBV newborn vaccination coverage cost:$I_{1}(t_{j})=\left( 1+r \right)^{-t_{j}}W_{1}\mu_{r}(\beta+(1-\beta)u_{5}\left( t \right))(N-R_{u}-X_{u}-Y_{ub})$

Infection diagnosis (both HBV and HDV) and HBV adult vaccination cost:$I_{2}(t_{j})=\rho_{0}\rho_{4}(t_{j})\left( 1+r \right)^{-t_{j}}[W_{2}\left( X\left( t_{j} \right)+4Y_{b1u}\left( t_{j} \right){+4Y}_{b2u}\left( t_{j} \right)+4Y_{a2u}\left( t_{j} \right)+2Y_{bd1u}\left( t_{j} \right)+{2Y}_{bd2u}\left( t_{j} \right)+R\left( t_{j} \right) \right)+W_{3}X(t_{j})]$

Mono-infected HBV treatment cost: $I_{3}\left( t_{j} \right)= W_{4}{\left( 1+r \right)^{-t_{j}}[\rho}_{1}\left( t_{j} \right)Y_{b2}\left( t_{j} \right)+Y_{bT1}(t_{j})+Y_{bT2}(t_{j})+Y_{bT3}(t_{j})]$

Dual-infected HBV-HDV treatment cost:$I_{4}(t_{j})= W_{5}\left( 1+r \right)^{-t_{j}}[\rho_{2}(t_{j})(Y_{bd1}(t_{j})+Y_{bd2}(t_{j}))]$

Awareness programs cost: $I_{5}(t_{j})=W_{6}{\left( 1+r \right)^{-t_{j}}u}_{3}(t_{j})[N-R_{u}\left( t_{j} \right)-R_{v}\left( t_{j} \right)-R_{R}\left( t_{j} \right)]$

The combined cost of all interventions at a time point ‘$t_{j}$’ can be given by,

$$G\left( t_{j} \right)=I_{1}(t_{j})+I_{2}(t_{j})+I_{3}(t_{j})+I_{4}(t_{j})+I_{5}(t_{j})$$

Even after applying all the interventions, a number of people may still be infected with HBV and HDV (residual infections) at the end of the 10 year. The lifetime cost of residual infections at time $T$ ($I_{6}$) is calculated as follows [9],

$$I_{6}(T)= L_{BT}\left( 1+r \right)^{-T}[{Y_{ub}\left( T \right)+Y_{ud}(T)+Y}_{b1u}\left( T \right)+Y_{b1}\left( T \right)+Y_{b2u}\left( T \right)+Y_{b2}\left( T \right)+Y_{bF}\left( T \right)+Y_{a2u}\left( T \right)+Y_{bd1u}\left( T \right)+Y_{bd1}\left( T \right)+Y_{bd2u}\left( T \right)+Y_{bd2}\left( T \right)+Y_{bdF}\left( T \right)+Y_{bT1}(T)+Y_{bT2}(T)+Y_{bT3}(T)]$$

where, $L_{BT}$ represents the lifetime cost of a residual infection. Therefore, the total cost (sum of cost of residual infections and interventions cost) at the end of time interval $T$ is given by $C\left( T \right)= \sum_{j=0}^{T-1} [G(t_{j}) ]+I_{6}(T)$.

## **Section C: Simulation procedure**

Our objective is to minimize the total number of HBV and HDV infections as well as the number of deaths caused by both hepatitis viruses at the end of time$T$, under the constraint that interventions costs $G(t_{j})$ at any time does not excess the health budget$B(t_{j})$. Mathematically, we aim to minimize$Y_{ub}\left( T \right)+Y_{ud}\left( T \right)+(1+\nu_{b2})(Y_{b1u}\left( T \right)+Y_{b2u}\left( T \right)+Y_{b1}\left( T \right)+Y_{b2}\left( T \right)+Y_{a2u}\left( T \right)+Y_{bF}(T))+(1+\nu_{bd2})(Y_{bd1u}\left( T \right)+Y_{bd2u}\left( T \right)+Y_{bd1}\left( T \right)+Y_{bd2}\left( T \right)+Y_{bdF}(T))+Y_{bT1}(T)+Y_{bT2}(T)+Y_{bT3}(T)$, subject to $G\left( t_{j} \right)\leq B(t_{j})$ and $0\leq\rho_{i}\left( t_{j} \right)\leq1$, $i=1,2,3,4,5$, where $t_{j}$ is year $j$ after the commencement of the program $j=0,\ldots9$ [9].

When$\rho_{i}\left( t_{j} \right)=1$, the intervention is applied to the entire eligible population segment at time$t_{j}$ as the probability of every single individual in the eligible population of a particular intervention ($\rho_{i}$) is also 100%. The solutions to these optimal intervention problems are determined after discretizing the time interval into yearly time steps and solving the differential equations over each time step to determine a value of $H(T)$ for a given choice of the intervention values. The interventions are held constant on each time step and the optimal levels of the interventions over each year $\rho_{t}$ are calculated using Genetic Algorithm in MATLAB R2016b under the stated constraints [31]. Results associated with the minimal objective function were then reported in the main text of the manuscript. The structure of the genetic algorithm employed in this manuscript is borrowed from the ref [31] and can be described as follows,

1. We call a control individual with the terminology $\bar{u}=\left\{ \rho_{1},\rho_{2},\rho_{3},\rho_{4},\rho_{5} \right\}$
2. For year 1, we generate 50 control individuals of the current generation such that $G\left( t_{1} \right)\leq B(t_{1})$ and $G\left( t_{1} \right)\geq0.99B(t_{1})$
3. Next, we determine total HBV and HDV prevalence as well as death toll for each of the 50 control individuals, and this is referred to as the fitness of the control individuals and arrange the 50 control individuals in the order of their fitness
4. Next, we select 25 control individuals with the best fitness and pass it on to the next generation of individuals
5. For the remaining 25 control individuals in the next generation, we employed breeding and mutation technique. To obtain one such control individual, we pick 10 control individual randomly from the current generation of 50 control individuals and arrange them in terms of their fitness, and the one with the best fitness is used as parent. We then select 10 more randomly from 40 remaining control individuals of the current generation, then find the one with the best fitness, and refer to that as mother. We breed them and create a child with mixing ratio of 80:20 between father and mother. In simple terms, it means that $\rho_{i}$ from father and mother is selected at 80% and 20% probability, respectively. If this new child do not satisfy the constraint, we induce mutation in it to satisfy the constraint, which is explained below.
6. Mutation:– we iteratively update new child $v_{c}$ by randomly selecting intervention $i$ at each iteration and updating it as follows until it satisfies constraint such that $G\left( t_{1} \right)\leq B(t_{1})$ and $G\left( t_{1} \right)\geq0.99B(t_{1})$

$$new v_{ci}=old v_{ci}+N(0,1)*(1-old v_{ci}), ifG\left( t_{1} \right)<0.99B(t_{1})$$

$$=old v_{ci}-N\left( 0,1 \right)*(1-old v_{ci}), ifG\left( t_{1} \right)>B(t_{1})$$

Where, $N(0,1)$ generates a random number between 0 and 1.

1. Steps 2 to 6 are repeated until we achieve convergence for the current year, means same control individual provide best fitness in five consecutive iterations.
2. Steps 2 to 7 are repeated for the remaining years in the chronological order.

## **Section D: HBV and HDV epidemics in Brazil and the Amazon Basin**

We study HBV and HDV epidemics on different levels in Brazil, (i) national level (Brazil), (ii) state level (State of Acre), (iii) city level (Manaus and Eirunepé city), and (iv) municipality level (Lábrea Municipality).

1. National level (Brazil): Out of the total population (~225 million), ~23% belongs to the age group 0-14 (or children in the model) [32]. In children age group, HBsAg prevalence is 1.8% [33] while in adults, HBsAg prevalence in 0.6% [11, 34]. We assume HDV prevalence in HBV infected children (due to household transmission) in Brazil on a national level to be negligible while HDV prevalence in HBV infected adults was estimated to be ~8% [35, 36].
2. Municipality level (Lábrea Municipality): The population of the Lábrea municipality, which is a part of the state Amazonas, was ~45,000 in 2017 (Ref: <http://www.citypopulation.de/php/brazil-amazonas.php?adm2id=1302405>). The population distribution between adults and children is assumed similar to the national level. HBsAg prevalence in all age groups is 8% [28, 37, 38]. HDV prevalence in HBV infected children is 7.7%, while HDV prevalence in HBV infected adults is 15.2% [38].
3. City level (Manaus): The population of Manaus, which is a part of the state Amazonas, was ~2.2 million in 2017 (<http://www.citypopulation.de/php/brazil-amazonas.php?adm2id=1302603>). The population distribution between adults and children is assumed similar as at the national level. HBsAg prevalence in adults is 6%[34] while HBsAg prevalence in children is assumed to be the same as at the national level of 1.8%. HDV prevalence in HBV infected adults is 27% [35] while HDV prevalence in children was assumed to 7.7%, same as Lábrea Municipality, another region in the Amazon Basin.
4. City level (Eirunepé city): The population of Eirunepé city, which is a part of the state Amazonas, was ~35000 in 2017. The population distribution between adults and children is assumed similar as at the national level. HBsAg prevalence in adults is 4.7% [35] while HBsAg prevalence in children is assumed the same as at the national level of 1.8%. HDV prevalence in HBV infected adults is 47% [35] while HDV prevalence in children was assumed to 7.7%, same as Lábrea Municipality, another region in the Amazon Basin.
5. State level (State of Acre): The population of the State of Acre is ~0.83 million in 2017 (Ref: <http://www.citypopulation.de/Brazil-Acre.html>). The population distribution between adults and children is assumed similar as at the national level. HBsAg prevalence in adults is 3.3% [39] while HBsAg prevalence in children is assumed the same as at the national level of 1.8%. HDV prevalence in HBV infected adults is 65% [35, 36, 39] while HDV prevalence in children was assumed to 7.7%, same as Lábrea Municipality, another region in the Amazon Basin.

## **Section E: Current budget for HBV and HDV control in Brazil**

In 2004, a small budget of $13.6 million was allocated to counter hepatitis viruses [40] and since then, it has been growing approximately at a rate of 11% [41]. This yields a health budget of $52.8 million for hepatitis viruses in 2017. Approximately 30.3% of all hepatitis cases were HCV-related [42]. If the budget is assumed to be distributed among hepatitis viruses based on the number of cases, we have $36 million in 2017 to counter HBV and HDV epidemics in Brazil. In addition to the federal health budget, states also allocate budget to promote health and counter diseases [41]; however, unlike federal government, we do not know what proportion of the total health budget state governments spend to tackle hepatitis viruses alone.

Moreover, highly HBV and HDV endemic Amazon Basin receives less than 5% of health budget in Brazil (i.e., $10.15 million) [40]. It is of interest to note that Amazon Basin is widespread in the 7 northern states of Brazil [40]. Out of 7 northern states, only 22.5% population resides in the state of Amazonas (with a population of ~4 million). Thus, if we assume the health budget to be distributed according to the population size, we have $2.28 million in 2017 to tackle HBV and HDV epidemics in the state of Amazonas.

Mathematically, this annual budget is given by$B\left( t_{j} \right)=B\left( 0 \right)\left( 1+0.11 \right)^{t_{j}}$, where $B\left( 0 \right)=$$36 million in Brazil and $B\left( 0 \right)=$$2.28 million in the state of Amazonas in the year 2017.


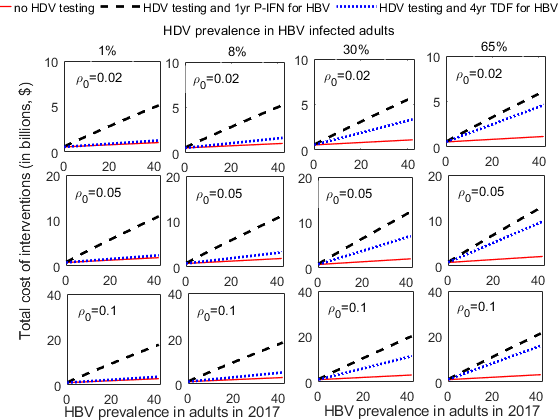


**Figure A. The total cost of implementing all interventions under different scenarios and unlimited resources.** The cost of implementing interventions over the next 10 years under: (i) No HDV testing with assuming all coinfections as mono-infections and treating them with 1-year of TDF therapy (red solid line), (ii) HDV testing and treating mono-infections with 4-years of TDF therapy while treating coinfections with 1-year of PEG-IFN therapy (blue dotted line), and (iii) HDV testing and treating mono-infections and co-infections with 1-year of PEG-IFN therapy (black dashed line).


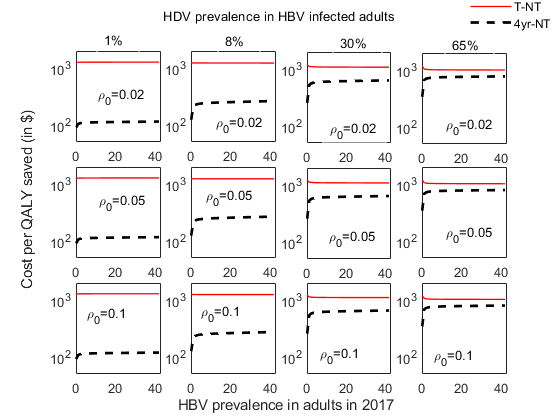


**Figure B. The cost per QALY saved (in $) under different scenarios and unlimited resources.** The maximum screening rate ($\rho_{0}$) and HDV prevalence in HBV mono-infected individuals are varied across columns and rows, respectively while the prevalence of HBV in the population is varied on the x-axis of each subfigure. The screening rate can be interpreted as approximately the proportion of the population that can be screened each year. The black dashed line shows the cost per QALY saved while comparing the case where HBV-only are treated with a 4-year course of TDF and co-infections with 1-year PEG-IFN to the case where there is no HDV testing and co-infections are treated similar to mono-infections with 1-year course of TDF. Similarly, the red line shows the cost per QALY saved while comparing the case where everyone is treated with PEG-IFN to the case where there is no HDV testing and co-infections are treated similar to mono-infections with 1-year course of TDF.

# References

1. Goyal A, Murray JM. Roadmap to control HBV and HDV epidemics in China. Journal of theoretical biology. 2017;423:41-52. doi: 10.1016/j.jtbi.2017.04.007. PubMed PMID: 28442239.

2. Ejidokun O, McNulty D, Linnane J, Ramaiah S. Sex education should begin in primary school. BMJ (Clinical research ed). 1999;318(7175):57. Epub 1999/01/05. PubMed PMID: 9872900; PubMed Central PMCID: PMCPMC1114550.

3. Niro GA, Fontana R, Ippolito AM, Andriulli A. Epidemiology and diagnosis of hepatitis D virus. Future Virology. 2012;7(7):709-17. doi: 10.2217/Fvl.12.61. PubMed PMID: WOS:000306149800014.

4. WorldBank. Life expectancy at birth, total (years): The World Bank; 2016. Available from: <http://data.worldbank.org/indicator/SP.DYN.LE00.IN?locations=BR>.

5. Goldstein ST, Zhou F, Hadler SC, Bell BP, Mast EE, Margolis HS. A mathematical model to estimate global hepatitis B disease burden and vaccination impact. International journal of epidemiology. 2005;34(6):1329-39. doi: 10.1093/ije/dyi206. PubMed PMID: 16249217.

6. Franco E, Bagnato B, Marino MG, Meleleo C, Serino L, Zaratti L. Hepatitis B: Epidemiology and prevention in developing countries. World J Hepatol. 2012;4(3):74-80. doi: 10.4254/wjh.v4.i3.74. PubMed PMID: 22489259; PubMed Central PMCID: PMCPMC3321493.

7. WHO. Guidelines for the prevention, care and treatment of persons with chronic hepatitis B infection. 2015.

8. Alexandre KV, Martins RM, Souza MM, Rodrigues IM, Teles SA. Brazilian hepatitis B vaccine: a six-year follow-up in adolescents. Mem Inst Oswaldo Cruz. 2012;107(8):1060-3. PubMed PMID: 23295759.

9. Goyal A, Murray JM. Recognizing the impact of endemic hepatitis D virus on hepatitis B virus eradication. Theor Popul Biol. 2016;112:60-9. doi: 10.1016/j.tpb.2016.08.004. PubMed PMID: 27594346.

10. Xiridou M, Borkent-Raven B, Hulshof J, Wallinga J. How hepatitis D virus can hinder the control of hepatitis B virus. PLoS One. 2009;4(4):e5247. Epub 2009/04/22. doi: 10.1371/journal.pone.0005247. PubMed PMID: 19381302; PubMed Central PMCID: PMCPMC2668760.

11. PAHO. Hepatitis B and C in the Spotlight. A public health response in the Americas, 2016. Washington, D.C, United States: 2016.

12. Luna EJ, Veras MA, Flannery B, de Moraes JC, Vaccine Coverage Survey G. Household survey of hepatitis B vaccine coverage among Brazilian children. Vaccine. 2009;27(39):5326-31. Epub 2009/07/21. doi: 10.1016/j.vaccine.2009.06.096. PubMed PMID: 19616495.

13. Goyal A, Murray JM. The impact of vaccination and antiviral therapy on hepatitis B and hepatitis D epidemiology. PLoS One. 2014;9(10):e110143. doi: 10.1371/journal.pone.0110143. PubMed PMID: 25313681; PubMed Central PMCID: PMCPMC4196970.

14. Paiva V, Calazans G, Venturi G, Dias R, Grupo de Estudos em Populacao SeA. [Age and condom use at first sexual intercourse of Brazilian adolescents]. Revista de saude publica. 2008;42 Suppl 1:45-53. Epub 2008/07/29. PubMed PMID: 18660924.

15. de Soarez PC, Sartori AM, de Andrade Lagoa Nobrega L, Itria A, Novaes HM. Cost-effectiveness analysis of a universal infant immunization program with meningococcal C conjugate vaccine in Brazil. Value in health : the journal of the International Society for Pharmacoeconomics and Outcomes Research. 2011;14(8):1019-27. doi: 10.1016/j.jval.2011.05.045. PubMed PMID: 22152170.

16. Eckman MH, Kaiser TE, Sherman KE. The cost-effectiveness of screening for chronic hepatitis B infection in the United States. Clin Infect Dis. 2011;52(11):1294-306. doi: 10.1093/cid/cir199. PubMed PMID: 21540206; PubMed Central PMCID: PMCPMC3097367.

17. Blatt CR, Bernardo NLMdC, Rosa JA, Bagatini F, Alexandre RF, Balbinotto Neto G, et al. An Estimate of the Cost of Hepatitis C Treatment for the Brazilian Health System. Value in Health Regional Issues. 2012;1(2):129-35. doi: 10.1016/j.vhri.2012.10.001.

18. Chen GF, Wei L, Chen J, Duan ZP, Dou XG, Xie Q, et al. Will Sofosbuvir/Ledipasvir (Harvoni) Be Cost-Effective and Affordable for Chinese Patients Infected with Hepatitis C Virus? An Economic Analysis Using Real-World Data. PLoS One. 2016;11(6):e0155934. doi: 10.1371/journal.pone.0155934. PubMed PMID: 27276081; PubMed Central PMCID: PMCPMC4898683.

19. Wiens A, Lenzi L, Venson R, Pedroso ML, Correr CJ, Pontarolo R. Economic evaluation of treatments for chronic hepatitis B. Braz J Infect Dis. 2013;17(4):418-26. doi: 10.1016/j.bjid.2012.12.005. PubMed PMID: 23849851.

20. Pham EA, Perumpail RB, Fram BJ, Glenn JS, Ahmed A, Gish RG. Future Therapy for Hepatitis B Virus: Role of Immunomodulators. Curr Hepatol Rep. 2016;15(4):237-44. doi: 10.1007/s11901-016-0315-9. PubMed PMID: 27917363; PubMed Central PMCID: PMCPMC5112294.

21. Ayoub WS, Keeffe EB. Review article: current antiviral therapy of chronic hepatitis B. Alimentary pharmacology & therapeutics. 2011;34(10):1145-58. doi: 10.1111/j.1365-2036.2011.04869.x. PubMed PMID: 21978243.

22. Terrault NA, Bzowej NH, Chang KM, Hwang JP, Jonas MM, Murad MH, et al. AASLD guidelines for treatment of chronic hepatitis B. Hepatology (Baltimore, Md). 2016;63(1):261-83. Epub 2015/11/14. doi: 10.1002/hep.28156. PubMed PMID: 26566064.

23. Heidrich B, Yurdaydin C, Kabacam G, Ratsch BA, Zachou K, Bremer B, et al. Late HDV RNA relapse after peginterferon alpha-based therapy of chronic hepatitis delta. Hepatology. 2014;60(1):87-97. Epub 2014/03/04. doi: 10.1002/hep.27102. PubMed PMID: 24585488.

24. Gringoes. Brazil News: Global Sex Survey 2017. Available from: <http://www.gringoes.com/brazil-news-global-sex-survey/>.

25. Barbosa RM, Koyama MAH. Comportamento e práticas sexuais de homens e mulheres, Brasil 1998 e 2005. Revista de saude publica. 2008;42:21-33.

26. Lok AS, Zoulim F, Dusheiko G, Ghany MG. Hepatitis B cure: From discovery to regulatory approval. Hepatology (Baltimore, Md). 2017;66(4):1296-313. doi: 10.1002/hep.29323. PubMed PMID: 28762522.

27. Ciancio A, Rizzetto M. Treatment of Hepatitis D. Viral Hepatitis: John Wiley & Sons, Ltd; 2013. p. 410-6.

28. Castilho Mda C, Oliveira CM, Gimaque JB, Leao JD, Braga WS. Epidemiology and molecular characterization of hepatitis B virus infection in isolated villages in the Western Brazilian Amazon. Am J Trop Med Hyg. 2012;87(4):768-74. doi: 10.4269/ajtmh.2012.12-0083. PubMed PMID: 22908032; PubMed Central PMCID: PMCPMC3516333.

29. Karahasanoglu FB, Asan A, Sacar S, Turgut H. Costs of treatment, follow-up, and complications of chronic hepatitis B and hepatitis C infections. Balkan medical journal. 2013;30(4):375-81. Epub 2014/09/11. doi: 10.5152/balkanmedj.2013.7547. PubMed PMID: 25207144; PubMed Central PMCID: PMCPMC4115953.

30. Wu B, Shen J, Cheng H. Cost-effectiveness analysis of different rescue therapies in patients with lamivudine-resistant chronic hepatitis B in China. BMC health services research. 2012;12:385. Epub 2012/11/10. doi: 10.1186/1472-6963-12-385. PubMed PMID: 23137013; PubMed Central PMCID: PMCPMC3511237.

31. Patel R, Longini IM, Jr., Halloran ME. Finding optimal vaccination strategies for pandemic influenza using genetic algorithms. Journal of theoretical biology. 2005;234(2):201-12. Epub 2005/03/11. doi: 10.1016/j.jtbi.2004.11.032. PubMed PMID: 15757679.

32. UN. World Population Prospects 2017 2017. Available from: <https://esa.un.org/unpd/wpp/Graphs/DemographicProfiles/>.

33. Villar LM, Amado LA, de Almeida AJ, de Paula VS, Lewis-Ximenez LL, Lampe E. Low prevalence of hepatitis B and C virus markers among children and adolescents. Biomed Res Int. 2014;2014:324638. doi: 10.1155/2014/324638. PubMed PMID: 25093164; PubMed Central PMCID: PMCPMC4100382.

34. Souto FJD. Distribution of hepatitis B infection in Brazil: the epidemiological situation at the beginning of the 21 st century. Revista da Sociedade Brasileira de Medicina Tropical. 2016;49:11-23.

35. Crispim MA, Fraiji NA, Campello SC, Schriefer NA, Stefani MM, Kiesslich D. Molecular epidemiology of hepatitis B and hepatitis delta viruses circulating in the Western Amazon region, North Brazil. BMC Infect Dis. 2014;14:94. Epub 2014/02/22. doi: 10.1186/1471-2334-14-94. PubMed PMID: 24555665; PubMed Central PMCID: PMCPMC3936897.

36. Elazar M, Koh C, Glenn JS. Hepatitis delta infection - Current and new treatment options. Best Pract Res Clin Gastroenterol. 2017;31(3):321-7. doi: 10.1016/j.bpg.2017.05.001. PubMed PMID: 28774414.

37. Braga WS, da Costa Castilho M, dos Santos IC, Moura MA, Segurado AC. Low prevalence of hepatitis B virus, hepatitis D virus and hepatitis C virus among patients with human immunodeficiency virus or acquired immunodeficiency syndrome in the Brazilian Amazon basin. Rev Soc Bras Med Trop. 2006;39(6):519-22. Epub 2007/02/20. PubMed PMID: 17308694.

38. Braga WS, Castilho Mda C, Borges FG, Leao JR, Martinho AC, Rodrigues IS, et al. Hepatitis D virus infection in the Western Brazilian Amazon - far from a vanishing disease. Rev Soc Bras Med Trop. 2012;45(6):691-5. PubMed PMID: 23295870.

39. Viana S, Parana R, Moreira RC, Compri AP, Macedo V. High prevalence of hepatitis B virus and hepatitis D virus in the western Brazilian Amazon. Am J Trop Med Hyg. 2005;73(4):808-14. Epub 2005/10/14. PubMed PMID: 16222030.

40. Parana R, Vitvitski L, Pereira JE. Hepatotropic viruses in the Brazilian Amazon: a health threat. The Brazilian journal of infectious diseases : an official publication of the Brazilian Society of Infectious Diseases. 2008;12(3):253-6. doi: <http://dx.doi.org/10.1590/S1413-86702008000300017>. PubMed PMID: 18833412.

41. Costa RM, Barbosa RS, Zucchi P. Expenditures in the health care system in Brazil: the participation of states and the Federal District in financing the health care system from 2002 to 2013. Clinics. 2015;70(4):237-41. doi: 10.6061/clinics/2015(04)03. PubMed PMID: PMC4418276.

42. Hanus JS, Ceretta LB, Simoes PW, Tuon L. Incidence of hepatitis C in Brazil. Rev Soc Bras Med Trop. 2015;48(6):665-73. doi: 10.1590/0037-8682-0230-2015. PubMed PMID: 26676490.
